# CT-based structural phenotypes of thoracic small-joint involvement in psoriatic arthritis

**DOI:** 10.3389/fmed.2026.1926072

**Published:** 2026-08-13

**Authors:** Tianshu Li, Zekun Zhang, Diancheng Li, Qian Tong, Dongdong Shen, Rui Li

**Affiliations:** 1 The First Orthopedics Department, Hebei Provincial Hospital of Traditional Chinese Medicine, Shijiazhuang, Hebei, China; 2 Department of Radiology, Hebei Provincial Hospital of Traditional Chinese Medicine, Shijiazhuang, Hebei, China

**Keywords:** ankylosis, arthritis, psoriasis, sternoclavicular joint, x-ray computed tomography

## Abstract

**Objective:**

This study sought to develop and evaluate an interpretable computed tomography (CT)-based structural phenotype framework for characterizing thoracic small-joint damage and differentiating CT-defined structural burden profiles in patients with PsA.

**Methods:**

In this retrospective study, 279 patients with PsA who underwent chest CT were included. The sternoclavicular joint (SCJ), costovertebral joint (CVJ), and costotransverse joint (CTJ) were systematically assessed for cortical erosion, joint-space narrowing, osteophyte formation, cortical sclerosis, and partial or complete ankylosis. A rule-based three-axis framework integrating destructive, proliferative, and locking lesions was constructed to classify patients into five mutually exclusive CT structural phenotypes. Upper-quartile CT structural burden was operationally defined using the cohort-specific 75th percentile as an internal stratification threshold.

**Results:**

Structural lesions were detected more frequently in the CVJ and CTJ than in the SCJ, with involvement rates of 83.2%, 84.6%, and 47.0%, respectively. The framework classified patients as low structural burden, destructive-dominant, proliferative-dominant, destructive–proliferative mixed, or locked phenotypes. Total structural burden differed significantly across phenotypes. Compared with the proliferative-dominant phenotype, the destructive–proliferative mixed and locked phenotypes were independently associated with upper-quartile CT structural burden after adjustment for age, sex, and disease duration, with adjusted odds ratios of 4.41 and 7.04, respectively.

**Conclusion:**

This CT-based structural phenotype framework offers a structured approach to describing thoracic small-joint damage and differentiating imaging-defined burden profiles.

## Introduction

Psoriatic disease extends beyond the skin and can involve multiple musculoskeletal compartments, including peripheral joints, the axial skeleton, entheses, and small articular structures that are often underrecognized in routine clinical assessment. Psoriatic arthritis (PsA) develops in a substantial proportion of patients with psoriasis and is characterized by marked clinical and imaging heterogeneity, which potentially leading to delayed recognition and incomplete characterization of structural damage (1, 2). Among its defining features, the coexistence of bone erosion and new bone formation distinguishes PsA from many other inflammatory arthritides and reflects the complex interaction between inflammation, aberrant bone remodeling, and mechanical stress (1, 3). Because structural damage represents cumulative disease impact and may persist beyond active inflammation, more precise imaging-based characterization is essential for understanding disease burden and improving communication between radiologists and rheumatologists.

Imaging plays a central role in the evaluation of PsA. Conventional radiography remains widely used for detecting established structural change, whereas magnetic resonance imaging and ultrasonography provide complementary information on active inflammation, enthesitis, and soft-tissue involvement (4). Computed tomography (CT), although not suitable for routine screening because of radiation exposure, provides high spatial resolution for cortical and periarticular bone abnormalities and is particularly useful in anatomically complex regions where radiographs are limited. However, most imaging studies in PsA have focused on peripheral joints, sacroiliac joints, or the spine, while the thoracic small-joint compartment has received comparatively limited systematic attention.

The sternoclavicular joint (SCJ), costovertebral joint (CVJ), and costotransverse joint (CTJ) are small but functionally important components of the thoracic skeleton, contributing to upper-limb linkage, rib–spine mechanics, and thoracic cage stability. Emerging evidence suggests that these joints may be clinically meaningful in inflammatory musculoskeletal disease. SCJ involvement in PsA has been described as an underrecognized manifestation associated with greater disease burden (5), while CT-based studies in axial spondyloarthritis have demonstrated that CVJ and CTJ structural lesions, including erosion, joint-space narrowing, osteophyte formation, and ankylosis, are detectable and may relate to structural progression or impaired mobility (6–8). These findings highlight thoracic small joints as an informative but insufficiently explored anatomical compartment in PsA.

Current approaches to thoracic joint assessment are often binary or descriptive and may not adequately capture the coexistence of destructive and proliferative processes characteristic of PsA. Isolated proliferative changes may overlap with degenerative remodeling, whereas combined erosion, joint-space alteration, proliferation, and ankylosis may better reflect disease-specific structural patterns. Despite growing interest in musculoskeletal imaging, no established CT-based phenotype framework has systematically integrated SCJ, CVJ, and CTJ lesions in PsA.

To address this gap, we developed a rule-based CT structural phenotype framework integrating destructive lesions, proliferative lesions, and ankylosis across these joints. We aimed to characterize lesion distribution, classify interpretable structural phenotypes, and evaluate their ability to identify upper-quartile CT structural burden beyond conventional approaches, hypothesizing that combined destructive–proliferative and ankylosing patterns represent more severe structural states.

## Methods

### Study design and participants

The retrospective study was approved by the Medical Ethics Committee of Hebei Provincial Hospital of Traditional Chinese Medicine (approval No. HBZY2026-KY-055-01). The requirement for informed consent was formally waived by the ethics committee. The study included patients with PsA who underwent clinically indicated chest CT examinations between January 2020 and December 2025. All CT examinations were obtained as part of routine clinical care and were not performed for research purposes or specifically for the assessment of PsA-related thoracic small-joint involvement. The diagnosis of PsA was based on the clinical diagnosis documented by rheumatologists in the contemporaneous medical records and was not inferred from the CT findings evaluated in this study.

Inclusion criteria were as follows: (1) rheumatologist-confirmed PsA fulfilling the Classification Criteria for Psoriatic Arthritis (CASPAR) criteria at baseline (9); (2) availability of chest CT images allowing assessment of the SCJ, CVJ, and CTJ; and (1) sufficient imaging quality and anatomical coverage for evaluation of the predefined target joints. Exclusion criteria were as follows: (1) poor-quality chest CT images that did not allow adequate assessment of the three target joints; (2) incomplete data for the three target joint sites; and (1) a history of chest surgery or other related treatments that could alter thoracic anatomical structures.

When multiple CT examinations were available for the same patient, the CT examination closest to the date of diagnosis was defined as the baseline examination and used for the main patient-level analysis, while additional CT examinations were not used to define the baseline phenotype.

Clinical and laboratory variables were extracted from medical records. Disease duration was calculated from the recorded onset or diagnosis date to the baseline CT examination, according to available clinical documentation. Laboratory variables were classified as elevated or positive according to the institutional laboratory reference ranges. The following adult cut-offs were applied: antistreptolysin O >200 IU/mL, procalcitonin ≥0.25 ng/mL, C-reactive protein >10 mg/L, rheumatoid factor >20 IU/mL, and antinuclear antibody positivity based on the reported antibody titer. Erythrocyte sedimentation rate was considered elevated according to age- and sex-specific reference limits. Composite PsA disease-activity indices, including the Disease Activity Index for Psoriatic Arthritis and the Psoriatic Arthritis Disease Activity Score, were not calculated because the required component variables were not systematically available within a predefined interval relative to the baseline CT examination. Standardized measurements of chest expansion, chest-wall pain, other patient-reported functional outcomes, and longitudinal clinical outcomes were also not routinely documented and were therefore not included in the analyses.

### Imaging acquisition and image review

Chest CT examinations were acquired as part of routine clinical care. Detailed CT acquisition parameters, including scanner type, tube voltage, tube current or automatic exposure settings, reconstruction algorithm, section thickness, reconstruction interval, window settings, use of contrast enhancement, and multiplanar reconstruction protocol, are summarized in Supplementary Table S1. The SCJ, CVJ, and CTJ were assessed as predefined thoracic small-joint compartments. The SCJ represented the anterior chest wall joint, whereas the CVJ and CTJ were considered posterior thoracic joints. CVJ assessment included bilateral joints from T1 to T12, yielding 24 costovertebral joints per patient. CTJ assessment included bilateral joints from T1 to T10, yielding 20 costotransverse joints per patient, because T11 and T12 do not form typical costotransverse joints. Bilateral SCJs were also assessed separately.

All CT findings were confirmed using multiplanar reconstructions. Two radiologists independently reviewed the CT images while blinded to clinical severity and phenotype classification. The original independent ratings obtained before consensus resolution were retained for inter-reader reliability analysis (Supplementary Table S2). Discrepant findings were subsequently resolved by joint review between the two radiologists, and the consensus assessments were used for the final study analyses.

Discrepant findings were resolved by joint review between the two radiologists, and the final classification was accepted only after consensus was reached.

### CT structural features

Five CT structural features were evaluated at the target joint sites: cortical erosion, joint-space narrowing, osteophyte formation, cortical sclerosis, and partial or complete ankylosis. Cortical erosion was defined as focal cortical interruption or irregular cortical bone loss at the articular surface. Joint-space narrowing was defined as definite narrowing, irregularity, or loss of the joint space. Osteophyte formation was defined as marginal or periarticular bony outgrowth. Cortical sclerosis was defined as increased subchondral bone density adjacent to the joint surface. Partial or complete ankylosis was defined as osseous bridging across part or all of the joint space.

For axis construction, each CT feature was coded as absent or present. Cortical erosion and joint-space narrowing were assigned to the destructive axis. Osteophyte formation and cortical sclerosis were assigned to the proliferative axis; however, these features may reflect both inflammatory new bone formation and degenerative remodeling. To minimize misclassification, sensitivity analyses excluding sclerosis were performed. Partial or complete ankylosis was assigned to the locking axis. When more than one feature was present in the same joint site, each feature was recorded separately.

### Structural burden scoring

**Figure 1:**
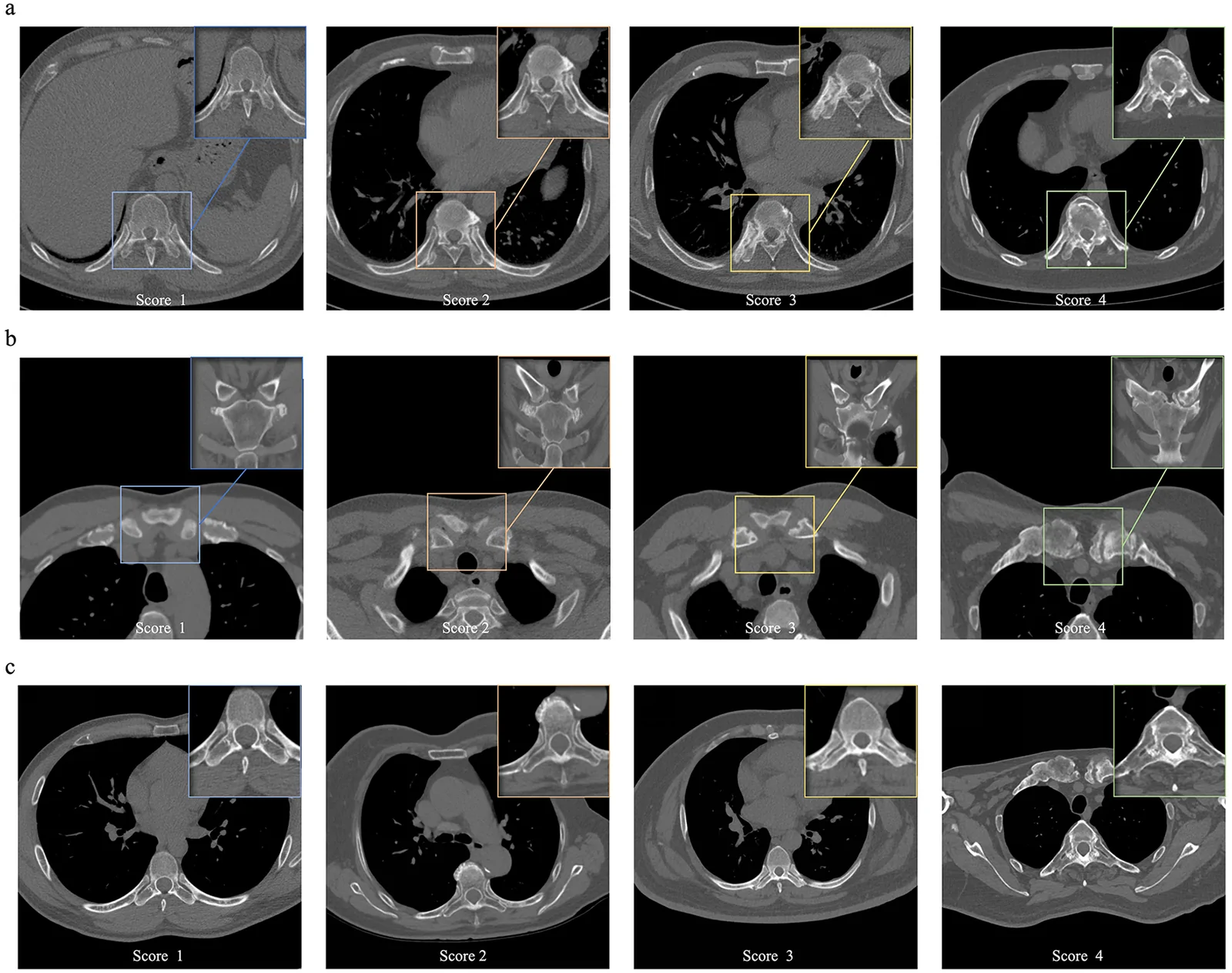
Representative CT features of structural damage according to severity score. Representative CT images illustrating structural severity scores 0–4 for the **(a)** costovertebral joint (CVJ), **(b)** sternoclavicular joint (SCJ), and **(c)** costotransverse joint (CTJ). In each panel, images are arranged from score 1 to score 4: score 1, suspicious structural abnormality; score 2, mild structural abnormality; score 3, moderate structural abnormality; and score 4, ankylosis. CVJ, costovertebral joint; SCJ, sternoclavicular joint; CTJ, costotransverse joint.

In addition to binary feature coding, structural burden was quantified using a semi-quantitative CT severity score (Figure 1). Each assessable CVJ and CTJ was graded from 0 to 4 according to the overall severity of structural damage: 0, normal; 1, suspicious abnormality; 2, mild structural abnormality, such as focal subtle erosion, sclerosis, or slight joint-space change; 3, moderate structural abnormality, such as definite subchondral bone destruction, marked joint-space narrowing, or articular irregularity; and 4, severe structural abnormality or complete ankylosis, characterized by osseous bridging across the joint space.

CVJ structural burden was calculated by summing the 0–4 scores of bilateral T1–T12 costovertebral joints, with a possible range of 0–96. CTJ structural burden was calculated by summing the 0–4 scores of bilateral T1–T10 costotransverse joints, with a possible range of 0–80. SCJ structural burden was calculated from bilateral SCJ scores using the same semi-quantitative principle. Left and right structural burden scores were calculated separately and then summed to obtain joint-compartment scores.

Total structural burden was calculated as the sum of SCJ, CVJ, and CTJ structural burden scores. Posterior structural burden was calculated as the sum of CVJ and CTJ structural burden scores. Positive joint sites were defined as the number of involved joint compartments among the SCJ, CVJ, and CTJ. For internal cohort stratification, upper-quartile CT structural burden was operationally defined as a total structural burden score at or above the cohort-specific 75th percentile (≥61 points). This data-derived threshold was used to distinguish patients with a relatively greater extent of CT-detectable structural involvement within the present cohort and was not intended to represent a clinically validated cutoff.

### CT-based three-axis structural phenotype classification

A rule-based three-axis CT structural phenotype model was constructed from the predefined binary CT features. At the patient level, the destructive axis was considered positive if cortical erosion or joint-space narrowing was present at any target joint site. The proliferative axis was considered positive if osteophyte formation or cortical sclerosis was present at any target joint site. The locking axis was considered positive if partial or complete ankylosis was present at any target joint site.

Patients were classified into five mutually exclusive phenotypes using a hierarchical rule. Patients with a positive locking axis were assigned to the locked phenotype, irrespective of destructive- or proliferative-axis status. Among patients without locking-axis positivity, those with negative destructive and proliferative axes were classified as low structural burden; those with a positive destructive axis and negative proliferative axis were classified as destructive-dominant; those with a positive proliferative axis and negative destructive axis were classified as proliferative-dominant; and those with both destructive- and proliferative-axis positivity were classified as destructive–proliferative mixed. Sensitivity analyses were conducted to assess the robustness of the phenotype framework, including age-stratified analyses using a 45-year cut-off, exclusion of degenerative-like proliferative-only cases, and repeated phenotype classification after removing cortical sclerosis from the proliferative axis.

### Statistical analysis

Continuous variables were summarized as mean ± standard deviation or median with interquartile range, and categorical variables as counts and percentages using non-missing denominators. Baseline characteristics across CT structural phenotype groups were compared using the Kruskal–Wallis test for continuous variables and the chi-square test for categorical variables. Paired comparisons of binary CT features and structural burden scores across SCJ, CVJ, and CTJ were performed using Cochran's Q test and the Friedman test, respectively. Inter-reader reliability was assessed using the two radiologists' independent pre-consensus evaluations; Cohen's kappa was used for binary CT features and patient-level classifications, quadratic-weighted kappa for regional ordinal severity grades, and a two-way random-effects, absolute-agreement, single-measure intraclass correlation coefficient for structural burden scores.

Logistic regression was used to assess the internal association between CT structural phenotype and upper-quartile CT structural burden, with the proliferative-dominant phenotype as the reference. Crude and adjusted odds ratios with 95% confidence intervals were calculated, adjusting for age, sex, and disease duration. Sensitivity analyses evaluated model robustness, including comparisons between locked and non-locked phenotypes. Additional age-stratified analyses were conducted to reduce potential confounding from degenerative joint changes. All tests were two-sided with *P* < 0.05 considered significant, and analyses were conducted using Python 3.11.7.

## Results

### Patient characteristics

All 279 patients (100%) had rheumatologist-confirmed psoriatic arthritis and fulfilled the CASPAR criteria at baseline (Table 1). The overall cohort had a mean age of 42.9 ± 16.0 years, included 210 males (75.3%), and had a median disease duration of 8.0 years (IQR, 2.0–16.0). According to the predefined CT structural phenotype classification, 37, 7, 72, 86, and 77 patients were classified into the low structural burden, destructive-dominant, proliferative-dominant, destructive–proliferative mixed, and locked phenotypes, respectively. Age differed significantly across the five phenotype groups, increasing from 21.0 ± 7.1 years in the low structural burden group to 54.6 ± 14.2 years in the locked group (*P* < 0.001). Disease-duration distributions differed across the five phenotype groups (*P* = 0.031; Table 1). Sex distribution did not differ significantly among phenotype groups (*P* = 0.129). None of the evaluated laboratory abnormalities differed significantly across the five CT structural phenotype groups, including elevated antistreptolysin O (*P* = 0.123), procalcitonin (*P* = 0.090), erythrocyte sedimentation rate (*P* = 0.808), and C-reactive protein (*P* = 0.246), as well as rheumatoid factor positivity (*P* = 0.689) and antinuclear antibody positivity (*P* = 0.265; Table 1).

**Table 1:** Baseline characteristics of the study cohort by CT structural phenotype.

Characteristic	Overall (*N* = 279)	Low structural burden (*n* = 37)	Destructive-dominant (*n* = 7)	Proliferative-dominant (*n* = 72)	Destructive–proliferative mixed (*n* = 86)	Locked (*n* = 77)	*P-value*
Age, years	42.9 ± 16.0	21.0 ± 7.1	31.4 ± 9.6	38.0 ± 9.8	47.0 ± 13.0	54.6 ± 14.2	< 0.001
Age < 45 years	160 (57.3)	37 (100.0)	6 (85.7)	57 (79.2)	42 (48.8)	18 (23.4)	< 0.001
Male sex	210 (75.3)	27 (73.0)	7 (100.0)	49 (68.1)	71 (82.6)	56 (72.7)	0.129
Disease duration, years	8.0 (2.0–16.0)	4.0 (1.0–8.0)	5.0 (4.0–10.0)	8.0 (2.8–10.2)	10.0 (2.2–20.0)	10.0 (2.0–20.0)	0.031
Antistreptolysin O elevated	7 (2.5)	2 (5.4)	1 (14.3)	2 (2.8)	2 (2.3)	0 (0.0)	0.123
Procalcitonin elevated	26 (9.3)	5 (13.5)	2 (28.6)	2 (2.8)	10 (11.6)	7 (9.1)	0.090
Erythrocyte sedimentation rate elevated	14 (5.0)	2 (5.4)	0 (0.0)	2 (2.8)	5 (5.8)	5 (6.5)	0.808
Rheumatoid factor positive	1 (0.4)	0 (0.0)	0 (0.0)	0 (0.0)	1 (1.2)	0 (0.0)	0.689
C-reactive protein elevated	14 (5.0)	1 (2.7)	1 (14.3)	1 (1.4)	7 (8.1)	4 (5.2)	0.246
Antinuclear antibody positive	50 (17.9)	5 (13.5)	0 (0.0)	10 (13.9)	16 (18.6)	19 (24.7)	0.265

Values are mean ± SD, median (interquartile range), or n (%). Percentages are calculated using non-missing denominators.

P-values compare the five phenotype groups using the Kruskal–Wallis test for continuous variables and the χ^2^ test for categorical variables.

### CT structural spectrum across target joints

**Figure 2:**
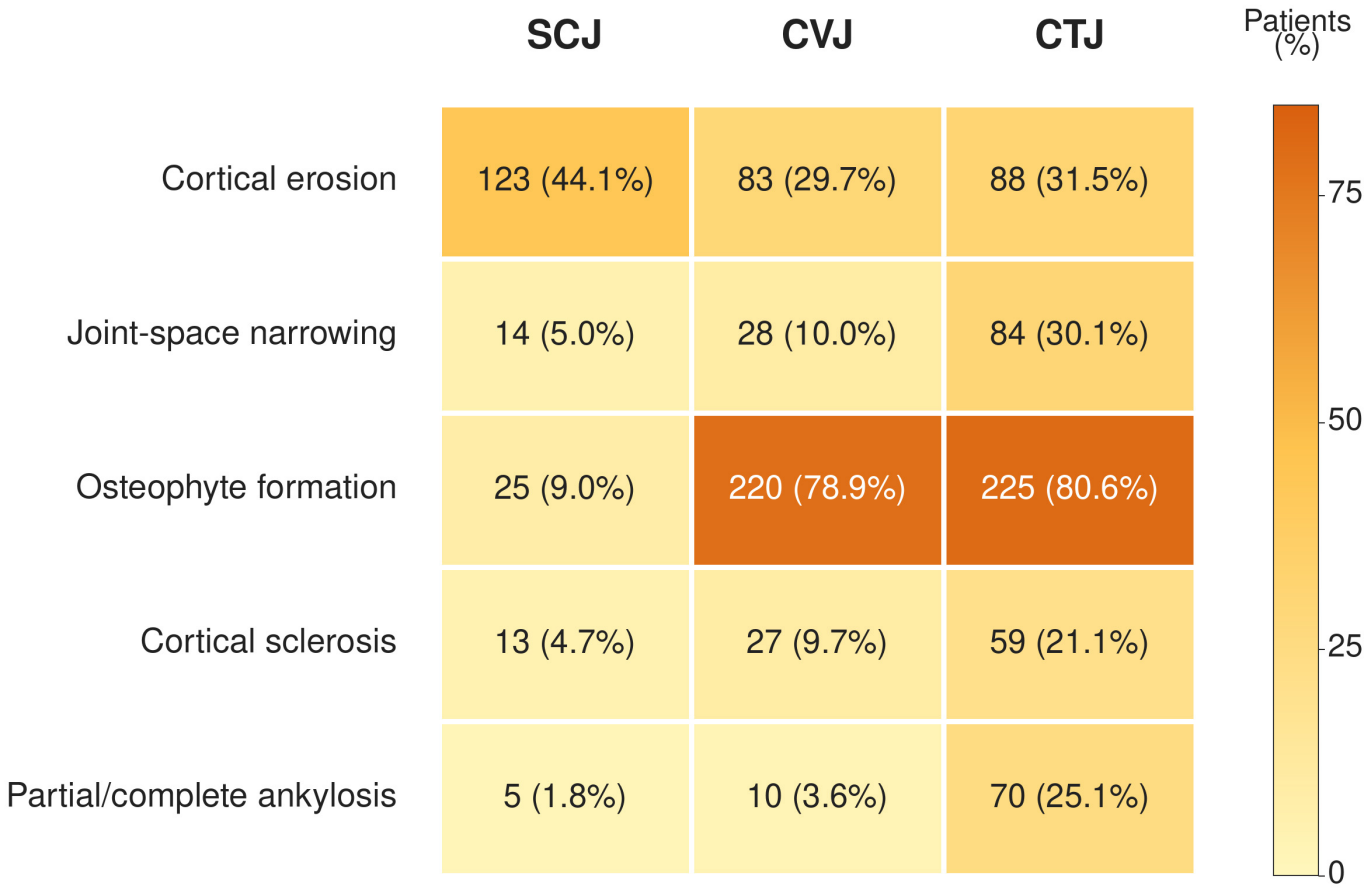
Joint-specific distribution of CT structural features heatmap showing the distribution of CT structural features across the sternoclavicular joint (SCJ), costovertebral joint (CVJ), and costotransverse joint (CTJ). Each cell presents the number and percentage of patients with the corresponding feature, shown as *n* (%). Color intensity represents the proportion of affected patients.

The distribution of CT structural lesions differed significantly among the SCJ, CVJ, and CTJ (Table 2, Figure 2). Any structural lesion was detected in 131 patients at the SCJ (47.0%), 232 patients at the CVJ (83.2%), and 236 patients at the CTJ (84.6%; *P* < 0.001). Cortical erosion was most frequently observed at the SCJ (123 patients, 44.1%), whereas osteophyte formation was predominantly observed at the CVJ (220 patients, 78.9%) and CTJ (225 patients, 80.6%). Partial or complete ankylosis was uncommon at the SCJ and CVJ, occurring in 5 patients (1.8%) and 10 patients (3.6%), respectively, but was substantially more frequent at the CTJ (70 patients, 25.1%; *P* < 0.001). Joint-level structural burden was also highest in the posterior thoracic joints, with median scores of 0.0 (IQR, 0.0–4.0), 26.0 (IQR, 12.0–34.0), and 18.0 (IQR, 10.0–26.0) for the SCJ, CVJ, and CTJ, respectively (*P* < 0.001).

**Table 2:** Distribution of CT structural features and joint-level structural scores across target joints.

CT feature or score	SCJ	CVJ	CTJ	Overall *P-value*
Any structural lesion	131 (47.0)	232 (83.2)	236 (84.6)	< 0.001
Cortical erosion	123 (44.1)	83 (29.7)	88 (31.5)	< 0.001
Joint space narrowing	14 (5.0)	28 (10.0)	84 (30.1)	< 0.001
Osteophyte formation	25 (9.0)	220 (78.9)	225 (80.6)	< 0.001
Cortical sclerosis	13 (4.7)	27 (9.7)	59 (21.1)	< 0.001
Partial or complete ankylosis	5 (1.8)	10 (3.6)	70 (25.1)	< 0.001
Destructive-axis score, *n* (%)				0.001
0	156 (55.9)	191 (68.5)	175 (62.7)	
1	109 (39.1)	65 (23.3)	36 (12.9)	
2	14 (5.0)	23 (8.2)	68 (24.4)	
Proliferative-axis score, *n* (%)				< 0.001
0	252 (90.3)	58 (20.8)	54 (19.4)	
1	16 (5.7)	195 (69.9)	166 (59.5)	
2	11 (3.9)	26 (9.3)	59 (21.1)	
Locking-axis score, *n* (%)				< 0.001
0	274 (98.2)	269 (96.4)	209 (74.9)	
1	5 (1.8)	10 (3.6)	70 (25.1)	
Left structural burden score	0.0 (0.0–2.0)	13.0 (6.0–18.0)	8.0 (4.0–14.0)	< 0.001
Right structural burden score	0.0 (0.0–2.0)	12.0 (6.0–16.0)	10.0 (6.0–14.0)	< 0.001
Joint structural burden score	0.0 (0.0–4.0)	26.0 (12.0–34.0)	18.0 (10.0–26.0)	< 0.001

Values are n (%) for binary CT features and median (interquartile range) for scores.

Overall P-values were calculated using Cochran's Q test for binary paired features and the Friedman test for paired joint scores.

SCJ, sternoclavicular joint; CVJ, costovertebral joint; CTJ, costotransverse joint.

### Rule-based CT structural phenotypes

**Figure 3:**
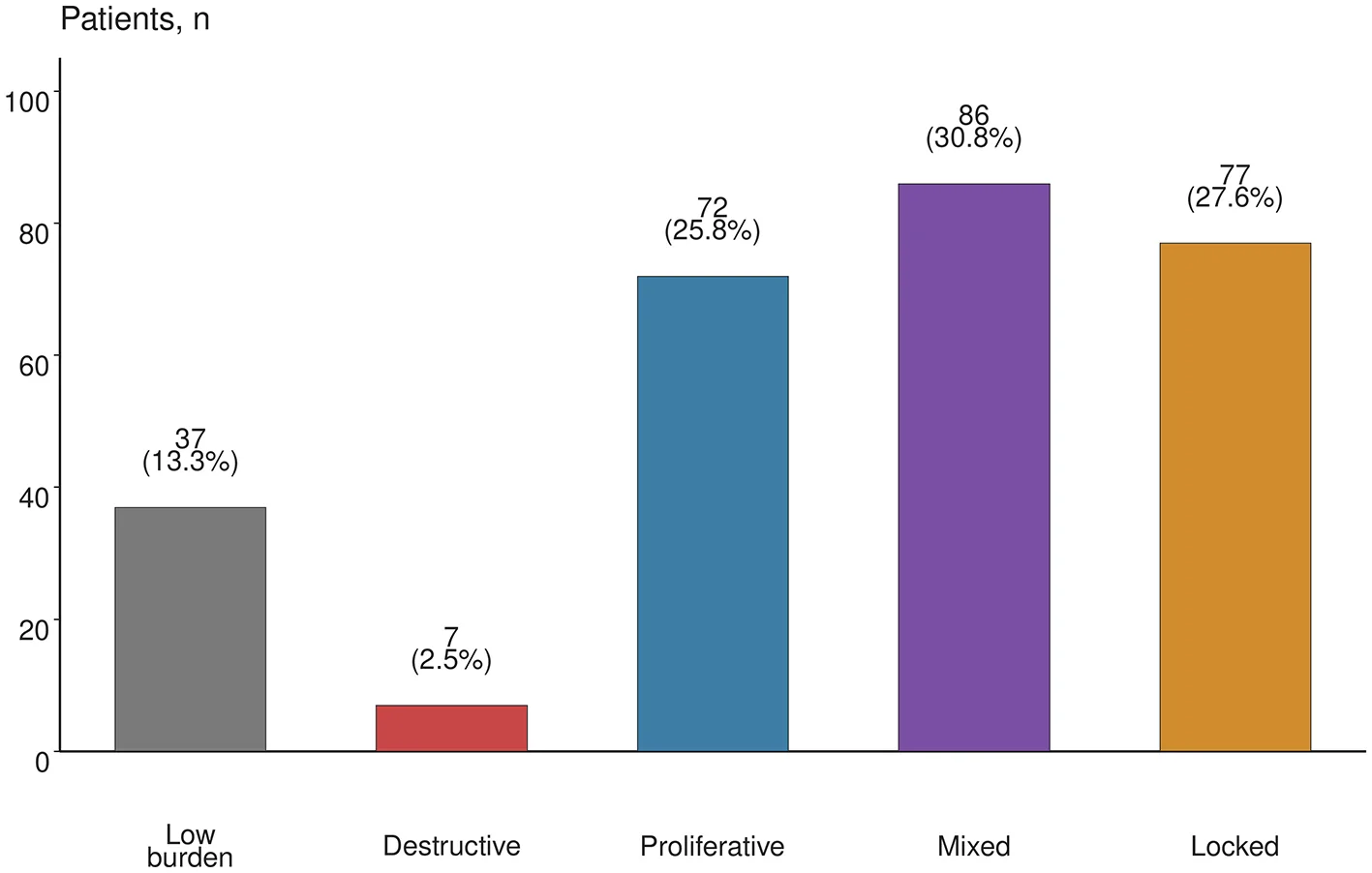
Distribution of CT structural phenotypes.

The rule-based three-axis classification identified five distinct CT structural phenotypes (Table 3, Figure 3). Among all patients, 37 (13.3%) had low structural burden, 7 (2.5%) had destructive-dominant disease, 72 (25.8%) had proliferative-dominant disease, 86 (30.8%) had destructive–proliferative mixed disease, and 77 (27.6%) had locked disease. Total structural burden differed significantly across the five phenotypes, with median scores of 0.0, 26.0, 35.0, 53.5, and 62.0, respectively (*P* < 0.001). A similar pattern was observed for posterior structural burden, which increased from 0.0 in the low structural burden phenotype to 58.0 in the locked phenotype (*P* < 0.001). The destructive-dominant, destructive–proliferative mixed and locked phenotypes also showed the broadest joint involvement, with a median of 3.0 positive joint sites in both groups.

**Table 3:** Rule-based CT structural phenotypes and structural burden across phenotype groups.

Structural phenotype	Rule-based definition	Patients, *n* (%)	Total structural burden	Posterior structural burden	SCJ score	CVJ score	CTJ score	Positive joint sites
Low structural burden	Destructive axis = 0; proliferative axis = 0; locking axis = 0	37 (13.3)	0.0 (0.0–0.0)	0.0 (0.0–0.0)	0.0 (0.0–0.0)	0.0 (0.0–0.0)	0.0 (0.0–0.0)	0.0 (0.0–0.0)
Destructive-dominant	Destructive axis > 0; proliferative axis = 0; locking axis = 0	7 (2.5)	26.0 (22.0–41.5)	26.0 (21.0–39.5)	1.0 (0.0–2.0)	16.0 (11.0–26.5)	12.0 (10.0–15.0)	3.0 (2.0–3.0)
Proliferative-dominant	Proliferative axis > 0; destructive axis = 0; locking axis=0	72 (25.8)	35.0 (23.5–46.5)	35.0 (23.5–46.0)	0.0 (0.0–0.0)	20.0 (12.0–26.0)	14.0 (10.0–22.0)	2.0 (2.0–2.0)
Destructive–proliferative mixed	Destructive axis > 0; proliferative axis > 0; locking axis = 0	86 (30.8)	53.5 (41.2–62.0)	52.0 (36.5–58.0)	4.0 (2.0–4.0)	29.5 (22.5–34.0)	20.0 (14.0–28.0)	3.0 (3.0–3.0)
Locked	Locking axis = 1, irrespective of destructive or proliferative axes	77 (27.6)	62.0 (48.0–71.0)	58.0 (48.0–67.0)	3.0 (0.0–4.0)	34.0 (28.0–36.0)	26.0 (19.0–32.0)	3.0 (2.0–3.0)
Overall *P-value*	Kruskal–Wallis test		< 0.001	< 0.001	< 0.001	< 0.001	< 0.001	< 0.001

Values are n (%) or median (interquartile range).

Total structural burden was calculated as the sum of SCJ, CVJ, and CTJ left/right scores. Posterior structural burden was calculated as CVJ plus CTJ scores.

The locked phenotype was assigned when the locking axis was positive, irrespective of destructive or proliferative-axis status.

### Association between CT structural phenotype and upper-quartile CT structural burden

**Figure 4:**
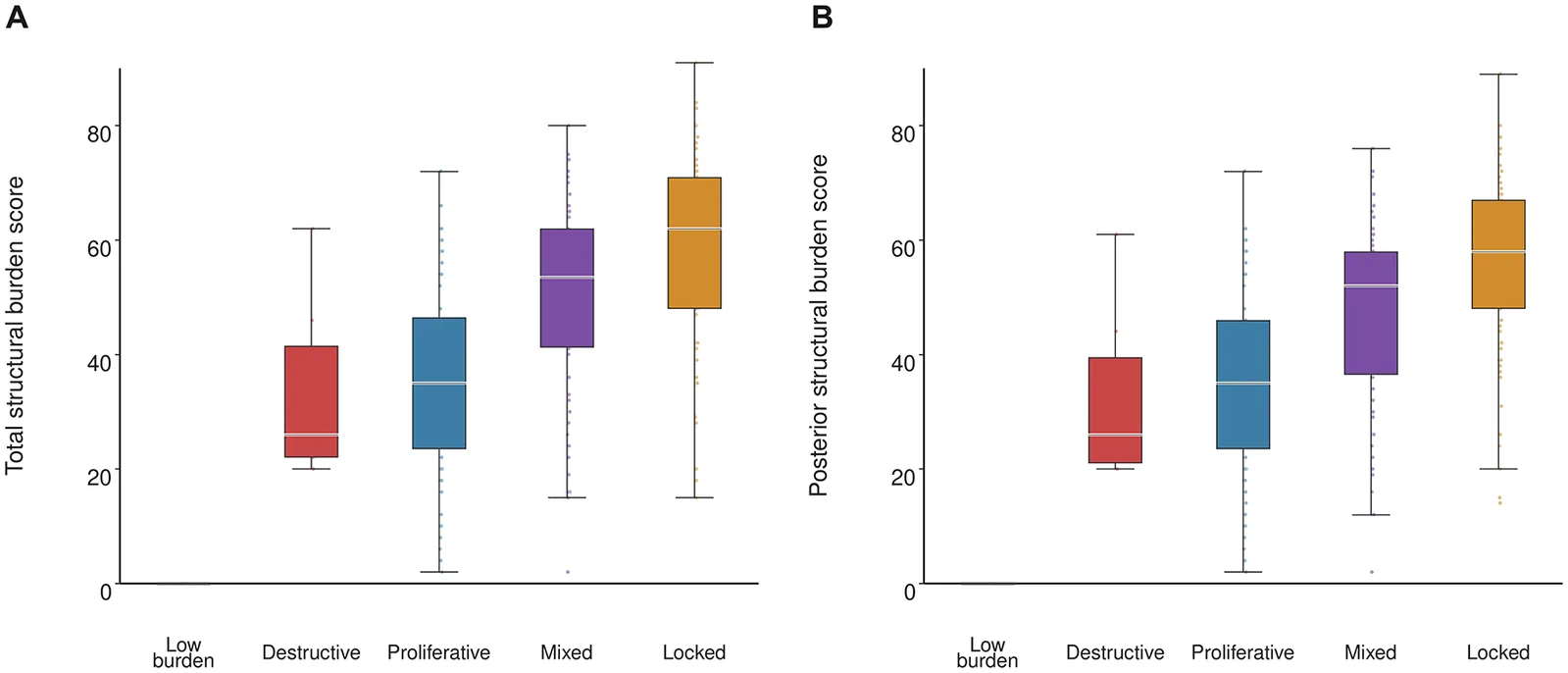
Structural burden across CT structural phenotypes. **(A)** Total structural burden score across phenotype groups. **(B)** Posterior structural burden score, calculated from the costovertebral and costotransverse joint compartments.

Upper-quartile CT structural burden was defined as a total structural burden score of at least 61 points, corresponding to the cohort 75th percentile (Table 4, Figure 4). Upper-quartile CT structural burden was observed in 0 of 37 patients (0.0%) with low structural burden, 1 of 7 patients (14.3%) with destructive-dominant phenotype, 4 of 72 patients (5.6%) with proliferative-dominant phenotype, 26 of 86 patients (30.2%) with destructive–proliferative mixed phenotype, and 40 of 77 patients (51.9%) with locked phenotype. Compared with the proliferative-dominant phenotype, the destructive–proliferative mixed phenotype and locked phenotype were significantly associated with upper-quartile CT structural burden in the crude analysis (OR = 7.37, 95% CI: 2.43–22.32, *P* < 0.001; and OR = 18.38, 95% CI: 6.10–55.38, *P* < 0.001, respectively). After adjustment for age, sex, and disease duration, these associations remained significant for both the destructive–proliferative mixed phenotype (adjusted OR = 4.41, 95% CI: 1.35–14.35, *P* = 0.014) and the locked phenotype (adjusted OR = 7.04, 95% CI: 2.12–23.36, *P* = 0.001).

**Table 4:** Association between CT structural phenotype and upper-quartile CT structural burden.

Structural phenotype	Patients, *n*	Upper-quartile CT structural burden, *n* (%)	Crude OR (95% CI)	Crude *P-value*	Adjusted OR (95% CI)	Adjusted *P-value*
Low structural burden	37	0 (0.0)	0.20 (0.01–3.87)	0.297	Not estimable	—
Destructive-dominant	7	1 (14.3)	2.83 (0.27–29.56)	0.379	6.09 (0.52–70.90)	0.149
Proliferative-dominant	72	4 (5.6)	1.00 (reference)	—	1.00 (reference)	—
Destructive–proliferative mixed	86	26 (30.2)	7.37 (2.43–22.32)	< 0.001	4.41 (1.35–14.35)	0.014
Locked	77	40 (51.9)	18.38 (6.10–55.38)	< 0.001	7.04 (2.12–23.36)	0.001

Upper-quartile CT structural burden was defined as total structural burden at or above the cohort 75th percentile (≥61 points).

Crude odds ratios compare each phenotype with the proliferative-dominant phenotype as the reference group.

Adjusted odds ratios were estimated using logistic regression adjusted for age, sex, and disease duration. The low-structural-burden phenotype was not estimable in the adjusted model because no high-burden events occurred in that group.

### Sensitivity analyses

Sensitivity analyses supported the robustness of the CT structural phenotype classification (Table 5). In the primary analysis, the locked phenotype was associated with a higher likelihood of upper-quartile CT structural burden than all non-locked phenotypes (OR = 5.96, 95% CI: 3.31–10.74, *P* < 0.001). After excluding degenerative-like proliferative-only cases, the locked phenotype remained significantly associated with upper-quartile CT structural burden (OR = 4.12, 95% CI: 2.23–7.64, *P* < 0.001). When sclerosis was removed from the proliferative axis, the phenotype distribution changed minimally, with 37 patients (13.3%) classified as low structural burden, 8 (2.9%) as destructive-dominant, 72 (25.8%) as proliferative-dominant, 85 (30.5%) as destructive–proliferative mixed, and 77 (27.6%) as locked; the association between the locked phenotype and upper-quartile CT structural burden remained significant (OR = 5.96, 95% CI: 3.31–10.74, *P* < 0.001).

**Table 5:** Sensitivity analyses of phenotype classification and upper-quartile CT structural burden.

Sensitivity analysis	Definition	*N*	Low structural burden, *n* (%)	Destructive-dominant, *n* (%)	Proliferative-dominant, *n* (%)	Destructive–proliferative mixed, *n* (%)	Locked, *n* (%)	Global P for total burden	High burden in locked phenotype	Locked vs. non-locked OR (95% CI)	*P-value*
Primary analysis	Original three-axis rule	279	37 (13.3)	7 (2.5)	72 (25.8)	86 (30.8)	77 (27.6)	< 0.001	40/77 (51.9)	5.96 (3.31–10.74)	< 0.001
Age < 45 years	Original rule, age-stratified subgroup	160	37 (23.1)	6 (3.8)	57 (35.6)	42 (26.2)	18 (11.2)	< 0.001	3/18 (16.7)	2.17 (0.55–8.56)	0.381
Age ≥45 years	Original rule, age-stratified subgroup	119	0 (0.0)	1 (0.8)	15 (12.6)	44 (37.0)	59 (49.6)	< 0.001	37/59 (62.7)	3.63 (1.70–7.74)	< 0.001
Degenerative-like cases excluded	Proliferative-only cases excluded	207	37 (17.9)	7 (3.4)	0 (0.0)	86 (41.5)	77 (37.2)	< 0.001	40/77 (51.9)	4.12 (2.23–7.64)	< 0.001
Sclerosis removed from proliferative axis	Osteophyte-only proliferative axis	279	37 (13.3)	8 (2.9)	72 (25.8)	85 (30.5)	77 (27.6)	< 0.001	40/77 (51.9)	5.96 (3.31–10.74)	< 0.001

Upper-quartile CT structural burden was defined using the primary cohort cut-off of ≥61 points.

Phenotype distributions are shown as n (%). Global P-values compare total structural burden across phenotypes using the Kruskal–Wallis test.

Odds ratios compare the locked phenotype with all non-locked phenotypes. The degenerative-like exclusion removed proliferative-only cases without erosion, narrowing, or ankylosis.

## Discussion

In this CT-based cohort of 279 patients, thoracic small-joint structural damage showed a highly heterogeneous but non-random distribution across the SCJ, CVJ, and CTJ. By integrating destructive, proliferative, and ankylosing lesions into a rule-based phenotype framework, we identified five structural phenotypes, among which the destructive–proliferative mixed and locked phenotypes were independently associated with upper-quartile CT structural burden after adjustment for age, sex, and disease duration.

A key finding of this study is that structural damage was not evenly distributed across the three target joints. Lesions were substantially more frequent in the posterior thoracic joints than in the SCJ, with any structural abnormality detected in more than 80% of patients at the CVJ and CTJ but in fewer than half at the SCJ. This pattern suggests that posterior rib–vertebral articulations represent the dominant anatomical compartment for CT-detectable structural involvement in this cohort. The lesion composition also differed by site: cortical erosion was most prominent at the SCJ, whereas osteophyte formation dominated at the CVJ and CTJ, and ankylosis was concentrated at the CTJ. These findings support the concept that anterior and posterior thoracic small joints may reflect different structural expressions of the same disease spectrum (6, 8). The SCJ may be more sensitive to erosive articular damage, whereas the CVJ and CTJ appear to accumulate proliferative and locking changes, possibly because of their role in thoracic cage stability and rib–spine mechanical transmission (6, 8).

The phenotype distribution further demonstrates that a simple binary definition of thoracic joint involvement is insufficient. Although proliferative-dominant disease accounted for approximately one quarter of the cohort, only a small proportion of these patients met the threshold for upper-quartile CT structural burden. In contrast, destructive–proliferative mixed and locked phenotypes accounted for most patients with advanced structural damage. This distinction is structurally informative because osteophyte formation alone may overlap with age-related or mechanical remodeling, whereas the coexistence of destructive and proliferative abnormalities may represent a more complex CT-defined pattern (1, 4). Therefore, the mixed phenotype may represent a more disease-informative structural pattern than proliferative change alone.

The locked phenotype showed the greatest CT-defined structural burden within the present cohort. More than half of patients with this phenotype had upper-quartile CT structural burden, and the association remained significant after adjustment for age, sex, and disease duration. This finding indicates that locking remained significantly associated with upper-quartile CT structural burden after adjusting for age or longer disease duration, although both age and disease duration differed across phenotype groups. Instead, the presence of partial or complete ankylosis identifies a distinct state of accumulated structural damage (7). The high posterior structural burden observed in the locked group is consistent with the anatomical distribution of ankylosis, which was uncommon at the SCJ and CVJ but frequent at the CTJ. Prior CT-based studies in axial spondyloarthritis have also highlighted the relevance of costovertebral and costotransverse joint involvement and have linked posterior thoracic ankylosis to structural progression and impaired spinal mobility (6, 7). Our findings extend this structural concept to a psoriasis-associated thoracic CT cohort and emphasize that CTJ locking may be a major contributor to overall thoracic structural burden.

The destructive–proliferative mixed phenotype deserves particular attention. Compared with the proliferative-dominant phenotype, the mixed phenotype had a markedly higher likelihood of upper-quartile CT structural burden in both crude and adjusted analyses. This result suggests that the addition of destructive lesions to proliferative remodeling changes the meaning of the imaging pattern: it no longer represents isolated bone formation but a combined structural process. Importantly, this association persisted after controlling for disease duration, indicating that mixed damage cannot be explained solely as a later stage of disease. Instead, it may reflect a combined structural pattern characterized by the coexistence of joint-surface damage and proliferative bone remodeling. This interpretation is consistent with current models of psoriatic arthritis pathophysiology, in which inflammatory pathways at the synovio-entheseal complex can drive both erosion and new bone formation (3, 4).

The robustness of the phenotype framework was supported by the sensitivity analyses. Excluding degenerative-like proliferative-only cases did not abolish the association between the locked phenotype and upper-quartile CT structural burden and removing sclerosis from the proliferative axis produced minimal changes in phenotype distribution. These analyses are important because proliferative CT findings, particularly osteophytes and sclerosis, can be difficult to distinguish from degenerative remodeling in an older population. The persistence of the main association after these alternative definitions indicates that the structural signal was not driven simply by age-related proliferative change. This strengthens the validity of separating proliferative-dominant disease from mixed and locked phenotypes, especially when applying the model to real-world CT datasets where degenerative and inflammatory structural findings may coexist (3, 4).

Compared with previous literature, this study adds several elements. Existing psoriatic arthritis studies have emphasized that structural damage involves both erosion and new bone formation, but most imaging work has focused on peripheral joints, sacroiliac joints, or the spine rather than systematically characterizing thoracic small joints (1, 2, 4). Recent evidence suggests that SCJ involvement in psoriatic arthritis may mark a more severe disease phenotype, but available data have been mainly clinical or focused on joint involvement rather than CT-defined structural patterns (5). In parallel, CT studies in spondyloarthritis have shown that CVJ and CTJ lesions are detectable and clinically meaningful (7, 8), but they have not established a phenotype framework integrating SCJ, CVJ, and CTJ lesions in a psoriasis-associated population (4, 6, 7). The present study bridges these gaps by converting individual CT signs into a structured, interpretable classification that reflects both lesion type and structural burden.

From an imaging and methodological perspective, thoracic small-joint CT findings may be more informatively described as combined structural patterns rather than as isolated abnormalities. The proposed framework provides a concise and interpretable vocabulary for distinguishing limited structural involvement, predominantly destructive change, predominantly proliferative change, mixed structural remodeling, and joint locking. When clinically indicated chest CT examinations are already available, systematic assessment of the SCJ, CVJ, and CTJ may help identify otherwise underrecognized thoracic structural involvement. In particular, mixed or locked phenotypes and extensive posterior involvement may facilitate communication between radiologists and rheumatologists and prompt targeted evaluation of thoracic symptoms, functional limitation, and the patient's overall clinical status (7, 8). However, because the present study did not include sufficiently complete disease-activity, symptom, or thoracic-function data, the framework should not be interpreted as a clinically validated disease-severity classification or as a threshold for treatment decision-making (3, 4).

Several limitations should be acknowledged. First, this retrospective opportunistic study used clinically acquired chest CT examinations performed for heterogeneous indications, which were not consistently available in standardized form. Selection bias therefore cannot be excluded, and the observed lesion prevalence may not be generalizable to an unselected PsA population. Second, the absence of a non-PsA control group limits assessment of disease specificity, particularly for proliferative lesions, while the small destructive-dominant subgroup reduces estimate precision. Third, the cohort-specific 75th-percentile threshold was intended only for internal imaging-based stratification and was not clinically validated. Standardized disease-activity indices, temporally aligned inflammatory markers, thoracic symptoms and functional measures, and longitudinal outcomes were not systematically available. Reliable information on biologic therapy before baseline CT was also incomplete, leaving potential residual confounding. In addition, the manubriosternal and sternocostal joints were not included in the prespecified imaging protocol, limiting assessment of anterior chest-wall involvement. Finally, CT has limited ability to evaluate active inflammation. Prospective controlled studies incorporating standardized clinical, laboratory, functional, treatment, and longitudinal data are needed before this framework can be considered clinically validated or used to guide treatment decisions.

In conclusion, this study suggests that CT-detected thoracic small-joint damage in PsA can be categorized into structural phenotypes with differing burden profiles. The destructive–proliferative mixed and locked phenotypes were associated with higher structural burden, indicating that a lesion-type-based framework may provide additional information beyond conventional binary involvement or total-score approaches.

## Data Availability

The original contributions presented in the study are included in the article/Supplementary material, further inquiries can be directed to the corresponding author.
