# Supplementary material for: CT-based structural phenotypes of thoracic small-joint involvement in psoriatic arthritis
Source: Front Med (Lausanne). 2026 Aug 13;13:1926072. doi: 10.3389/fmed.2026.1926072 (PMC13519402; doi:10.3389/fmed.2026.1926072)
Supplement: Supplementary file 1 [file Data_Sheet_1.docx]

**Table S1. Chest CT acquisition and reconstruction parameters**

| **Parameter** | **GE Revolution CT** | **United Imaging 80-row CT** | **Siemens 64-row CT** |
| --- | --- | --- | --- |
| **Scanner hardware class** | Wide-detector multidetector CT; 16-cm z-axis detector coverage capability | 80-row multidetector CT platform | 64-row multidetector CT platform |
| **Patient position** | Supine; arms raised when feasible | Supine; arms raised when feasible | Supine; arms raised when feasible |
| **Scan coverage** | Thoracic inlet to costophrenic angles; SCJ, CVJ, and CTJ included | Thoracic inlet to costophrenic angles; SCJ, CVJ, and CTJ included | Thoracic inlet to costophrenic angles; SCJ, CVJ, and CTJ included |
| **Tube voltage protocol** | Routine chest protocol centered on 120 kVp; automatic kV selection allowed when available | Routine chest protocol centered on 120 kVp; automatic kV selection allowed when available | Routine chest protocol centered on 120 kVp; CARE kV/automatic kV selection allowed when available |
| **Tube current control** | Automatic exposure control (GE Smart mA/Auto mA); mA varied by patient habitus | Automatic exposure control; mA varied by patient habitus | Automatic exposure control (CARE Dose4D or equivalent); mA varied by patient habitus |
| **Detector/collimation setting** | Thin-section helical chest acquisition using Revolution CT detector configuration | Thin-section helical chest acquisition using 80-row detector configuration | Thin-section helical chest acquisition using 64-row detector configuration |
| **Rotation time setting** | Approximately 0.28-0.50 s according to chest protocol | Approximately 0.35-0.50 s according to chest protocol | Approximately 0.33-0.50 s according to chest protocol |
| **Pitch setting** | Approximately 0.8-1.2, protocol- and coverage-dependent | Approximately 0.8-1.2, protocol- and coverage-dependent | Approximately 0.8-1.2, protocol- and coverage-dependent |
| **Matrix / field of view** | 512 x 512; FOV adjusted to thoracic coverage and body habitus | 512 x 512; FOV adjusted to thoracic coverage and body habitus | 512 x 512; FOV adjusted to thoracic coverage and body habitus |
| **Routine image reconstruction** | Standard chest kernel with vendor-specific iterative reconstruction when used clinically | Standard chest kernel with vendor-specific iterative reconstruction when used clinically | Standard chest kernel with Siemens iterative reconstruction when used clinically |
| **Bone reconstruction kernel** | High-spatial-frequency bone/edge-enhancing kernel | High-spatial-frequency bone/sharp kernel | High-spatial-frequency bone kernel, such as B60f/B70f or equivalent |
| **Reconstructed section thickness** | 1.0 mm | 1.0 mm | 1.0 mm |
| **Reconstruction interval** | 1.0 mm or overlapping interval according to scanner protocol | 1.0 mm or overlapping interval according to scanner protocol | 1.0 mm or overlapping interval according to scanner protocol |
| **MPR review** | Axial, coronal, and sagittal 1.0-mm multiplanar reconstructions | Axial, coronal, and sagittal 1.0-mm multiplanar reconstructions | Axial, coronal, and sagittal 1.0-mm multiplanar reconstructions |
| **Window setting for scoring** | Bone window, WW/WL = 2000/500 HU | Bone window, WW/WL = 2000/500 HU | Bone window, WW/WL = 2000/500 HU |
| **Contrast enhancement** | Non-contrast or contrast-enhanced CT accepted; scoring used bone-window reconstructions | Non-contrast or contrast-enhanced CT accepted; scoring used bone-window reconstructions | Non-contrast or contrast-enhanced CT accepted; scoring used bone-window reconstructions |

**Note.** Values are presented as scanner-specific routine chest CT protocol settings or protocol ranges rather than fixed patient-level DICOM values. Tube current, pitch, and rotation time may vary between examinations because automatic exposure control and patient habitus were used in routine clinical scanning. For the present structural assessment, all target joints were reviewed on 1.0-mm thin-section multiplanar reconstructions using a bone window setting of WW/WL 2000/500 HU. SCJ, sternoclavicular joint; CVJ, costovertebral joint; CTJ, costotransverse joint; MPR, multiplanar reconstruction; WW, window width; WL, window level; HU, Hounsfield units.

**Table S2. Inter-reader reliability of CT structural features, severity scores, and phenotype classification**

| **Variable** | **N** | **Reader 1** | **Reader 2** | **Consensus / reference** | **Agreement, %** | **Reliability estimate** | **95% CI** |
| --- | --- | --- | --- | --- | --- | --- | --- |
| **A. Binary CT structural features** | | | | | | | |
| SCJ erosion | 279 | 121 (43.4%) | 124 (44.4%) | 123 (44.1%) | 96.1 | κ = 0.920 | 0.874–0.966 |
| SCJ joint-space narrowing | 279 | 13 (4.7%) | 15 (5.4%) | 14 (5.0%) | 98.6 | κ = 0.850 | 0.705–0.994 |
| SCJ osteophyte | 279 | 24 (8.6%) | 26 (9.3%) | 25 (9.0%) | 97.8 | κ = 0.868 | 0.765–0.972 |
| SCJ sclerosis | 279 | 12 (4.3%) | 14 (5.0%) | 13 (4.7%) | 98.6 | κ = 0.839 | 0.684–0.994 |
| SCJ ankylosis | 279 | 5 (1.8%) | 6 (2.2%) | 5 (1.8%) | 99.6 | κ = 0.907 | 0.727–1.000 |
| CVJ erosion | 279 | 81 (29.0%) | 84 (30.1%) | 83 (29.7%) | 96.1 | κ = 0.905 | 0.851–0.960 |
| CVJ joint-space narrowing | 279 | 27 (9.7%) | 29 (10.4%) | 28 (10.0%) | 97.8 | κ = 0.881 | 0.787–0.975 |
| CVJ osteophyte | 279 | 218 (78.1%) | 221 (79.2%) | 220 (78.9%) | 96.8 | κ = 0.904 | 0.842–0.966 |
| CVJ sclerosis | 279 | 26 (9.3%) | 28 (10.0%) | 27 (9.7%) | 97.8 | κ = 0.877 | 0.780–0.974 |
| CVJ ankylosis | 279 | 9 (3.2%) | 11 (3.9%) | 10 (3.6%) | 98.6 | κ = 0.793 | 0.595–0.990 |
| CTJ erosion | 279 | 87 (31.2%) | 90 (32.3%) | 88 (31.5%) | 96.1 | κ = 0.909 | 0.856–0.962 |
| CTJ joint-space narrowing | 279 | 83 (29.7%) | 86 (30.8%) | 84 (30.1%) | 96.1 | κ = 0.907 | 0.853–0.961 |
| CTJ osteophyte | 279 | 223 (79.9%) | 226 (81.0%) | 225 (80.6%) | 96.1 | κ = 0.875 | 0.802–0.947 |
| CTJ sclerosis | 279 | 58 (20.8%) | 61 (21.9%) | 59 (21.1%) | 96.8 | κ = 0.904 | 0.842–0.966 |
| CTJ ankylosis | 279 | 68 (24.4%) | 71 (25.4%) | 70 (25.1%) | 96.8 | κ = 0.914 | 0.858–0.969 |
| **B. Patient-level structural burden scores** | | | | | | | |
| SCJ structural burden score | 279 | 2.0 ± 2.4 | 2.0 ± 2.3 | 0.0 (0.0–4.0) | — | ICC = 0.935 | 0.916–0.949 |
| CVJ structural burden score | 279 | 25.1 ± 17.8 | 25.3 ± 17.4 | 26.0 (12.0–34.0) | — | ICC = 0.970 | 0.963–0.976 |
| CTJ structural burden score | 279 | 18.9 ± 13.7 | 19.3 ± 13.9 | 18.0 (10.0–26.0) | — | ICC = 0.950 | 0.936–0.962 |
| Posterior structural burden score | 279 | 44.0 ± 25.7 | 44.5 ± 25.5 | 42.0 (25.5–58.0)† | — | ICC = 0.972 | 0.966–0.978 |
| Total structural burden score | 279 | 46.0 ± 25.9 | 46.5 ± 25.7 | 43.0 (28.0–61.0)† | — | ICC = 0.972 | 0.965–0.978 |
| **C. Patient-level classifications** | | | | | | | |
| Five-category structural phenotype | 279 | 38/8/71/85/77 | 36/8/73/85/77 | 37/7/72/86/77 | 92.8 | κ = 0.904 | 0.863–0.944 |
| Upper-quartile structural burden, ≥61 points | 279 | 72 (25.8%) | 73 (26.2%) | 71 (25.4%) | 96.8 | κ = 0.916 | 0.862–0.970 |

**Notes:** For binary features, Reader 1 and Reader 2 results are positive counts with percentages. For ordinal grades, results are distributions across grades 0/1/2/3/4. For burden scores, results are mean +/- standard deviation. For the five-category phenotype, distributions are shown in the order low-burden/destructive/proliferative/mixed/locked. Binary features and patient-level classifications were evaluated using Cohen's kappa; ordinal grades were evaluated using quadratic-weighted Cohen's kappa; burden scores were evaluated using a two-way random-effects, absolute-agreement, single-measure intraclass correlation coefficient.

**Abbreviations:** CI, confidence interval; CTJ, costotransverse joint; CVJ, costovertebral joint; ICC, intraclass correlation coefficient; SCJ, sternoclavicular joint.
